# Link Prediction in Evolving Networks Based on Popularity of Nodes

**DOI:** 10.1038/s41598-017-07315-4

**Published:** 2017-08-02

**Authors:** Tong Wang, Xing-Sheng He, Ming-Yang Zhou, Zhong-Qian Fu

**Affiliations:** 1 Department of Electronic Science and Technology, University of Science and Technology of China, Hefei, 230027 P. R. China; 2 Guangdong Province Key Laboratory of Popular High Performance Computers, College of Computer Science and Software Engineering, Shenzhen University, Shenzhen, 518060 P. R. China; 3 Physics Department, University of Fribourg, Chemin du Musée 3, Fribourg, CH-1700 Switzerland

## Abstract

Link prediction aims to uncover the underlying relationship behind networks, which could be utilized to predict missing edges or identify the spurious edges. The key issue of link prediction is to estimate the likelihood of potential links in networks. Most classical static-structure based methods ignore the temporal aspects of networks, limited by the time-varying features, such approaches perform poorly in evolving networks. In this paper, we propose a hypothesis that the ability of each node to attract links depends not only on its structural importance, but also on its current popularity (activeness), since active nodes have much more probability to attract future links. Then a novel approach named popularity based structural perturbation method (PBSPM) and its fast algorithm are proposed to characterize the likelihood of an edge from both existing connectivity structure and current popularity of its two endpoints. Experiments on six evolving networks show that the proposed methods outperform state-of-the-art methods in accuracy and robustness. Besides, visual results and statistical analysis reveal that the proposed methods are inclined to predict future edges between active nodes, rather than edges between inactive nodes.

## Introduction

Networks are effective descriptions of complex systems in society and nature^1, 2^, with entities denoted as nodes and relations as links, respectively. The organization of real networks evolve under the influence of certain patterns and irregular factors, in principle, only the former can be modeled with physical methodologies. A significant concern about complex networks is link prediction that conduces to explanations of these models and revelations of the hidden driving-mechanisms. Therefore, link prediction has drawn numerous attentions from various fields covering biology, sociology and others^3–6^. For example, in protein-protein interaction experiments in cells, only strong relations between proteins could be detected by limited precision of equipments. It is prohibitive to measure every interaction between all pair proteins due to sharply increasing experimental costs with the size of proteins^7, 8^, an appropriate approach is to evaluate the likelihood of potential relations and specifically test non-existing relations with the high likelihood. Also, in social contexts, two persons would build friendship in the near future with a high probability if they have many common friends or attributes, which could be utilized to uncover lost friends or predict future friends^9–11^. Besides, further extensive applications also include personalized recommendations in e-commerce^12, 13^ and aircraft route planning study^14^, etc.

The crux of link prediction is to evaluate the likelihood of potential edges, based on which we can rank the potential edges in descending order and edges in the top of ranking list are predicted as underlying or future edges^15, 16^. The similarity based approaches, which equate likelihood with similarity, are the most common frameworks that argue the prospective edges may exist between similar nodes. To achieve this, traditional attribute based methods measure the likelihood of links by learning how many common features (e.g. common hobbies, ages, tastes, geographical locations) the two endpoints share^17^. Many researches on social networks have shown that the pervasive homophily promotes ties between similar humans^18, 19^. However this kind of methods suffer from the inaccessible and unreliable information of nodes due to the privacy policy in real scenario^20^. Luckily, the development of the complex network theory provides a new path in which only network topological structure is required regardless of privacy information to solve the problem. When evaluating the similarity between nodes, according to the structure differences, structure based methods could be classified into three categories: local methods, global methods and Quasi-global methods. Local similarity is mainly based on common neighbors, such as the most well-known Common Neighbor (CN) index that counts the number of common neighbor nodes^21^, Adamic-Adar (AA) index and Resource Allocation (RA) index that depress the large-degree neighbor nodes^22, 23^. For large networks, Cui *et al*. proposed a fast algorithm for calculating the number of common neighbors^24^. Global similarity emphasizes the global topology information of network, such as Katz index that counts all of the paths between two nodes^25^. Quasi-global similarity is a well trade-off of local similarity methods and global similarity methods, such as Local Path (LP) index that only considers the short paths in Katz index^23^, Local Random Walk (LRW) index that focuses on the limited random walk in local area^26^. Beyond that, some algorithms based on maximum likelihood methods and other exquisite models have been proposed. Clauset *et al*. proposed a Hierarchical Structure Model which presents well performance in hierarchical networks by using a dendrogram^27^. Lü *et al*. proposed a Structural Perturbation Method that approximates the observed networks by randomly repeated perturbations. This method outperforms state-of-the-art methods in accuracy and robustness^28^. In terms of information theory, Xu *et al*. proposed the Path Entropy index that considers the information entropies of shortest paths and penalizes the long paths^29^. Tan *et al*. proposed a Mutual Information (MI) method with the high accuracy and reasonable computation time, which considers the feature of common neighbors and denotes the likelihood of one link as the the conditional self-information of this link existing between the node pair when their common neighbors are given^30^. Zhu *et al*. generalized the MI index into Neighbor Set Information that is applicable to multiple structural features to enhance the accuracy^31^.

**Figure 1:**
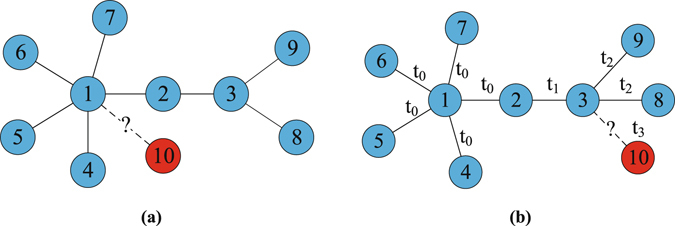
Illustration of the popularity. The fresh link and node 10 will be added into the existing networks at the next time *t*_*3*_. In panel (a), attractiveness of nodes are determined by static features. According to the preferential attachment, node 10 prefers to connect with node 1 due to the largest degree. In panel (b), temporal effects are considered. The currently popular node 3 may become attractive and connect with node 10 at time *t*_3_.

Real networks are highly dynamic with the come-and-go of nodes and edges^32^. However, the aforementioned algorithms unexceptionally ignore the temporal aspects of real networks, in particular, the trend of nodes: yesterday active nodes that contacted numerous neighbors may be unpopular today. Inspired by this, we propose a hypothesis that the emergence of future links are not only determined by existing network structure, but also are affected by the popularity of endpoints. For instance, Fig. 1 illustrates the effects of popularity. The red node will enter in the network and connect with one of the existing nodes. In Fig. 1(a), according to the static analysis, node 10 prefers to connect with the large-degree node 1. While the birth time of each edge is given in Fig. 1(b), we can easily know that node 3 is of high popularity because only it attracts edges at the present time *t*_2_. In practice, the fresh edge will be more likely to occur between node 10 and the active node 3 at the next period *t*_3_. To comply with this scenario, unlike previous works that predict potential links mostly based on static networks, we propose a popularity based structural perturbation method (PBSPM) and its fast algorithm that integrate popularity of nodes and observed network topology to predict future edges. Experimental results on real-world networks show that the proposed methods outperform the other traditional approaches in accuracy and robustness.

## Results

### Popularity metrics

The definition of popularity is related to the concepts of temporal trend of nodes that could be obtained through the statistics and analysis of relevant historical information. For two nodes with the same degree, one may connect with its neighbors at early stage and not form any connections later, while the other one develops most of its connections at late stage. Intuitively the latter node would attract more fresh edges with high probability in the near future. Given this, a straightforward approach to evaluate the popularity of a node is counting the edges it recently attracts.

Given an undirected and unweighted network *G*(*V*, *E*) where *V* and *E* represent the set of nodes and links, respectively, each link has a time-stamp that represents the entering time. In this work, multi-links and self-loops are not allowed. *k*_*i*_(*t*) denotes the degree of nodes *i* at time *t*. In the next time span *T*, node *i* would attract Δ*k*_*i*_(*t*, *T*) new edges,

1 $${\rm{\Delta }}{k}_{i}(t,T)={k}_{i}(t+T)-{k}_{i}(t)\mathrm{.}$$

Note that Δ*k*_*i*_(*t*, *T*) in Eq. (1) determined by both *t* and *T* cannot reflect the relative popularity of node *i*, since even large degree nodes become inactive, they still attract more fresh edges than nodes of small degree due to the preferential attachment mechanism. To solve this issue, for a dataset spans starting from *t*_*a*_ to *t*_*c*_, we divide its edges into the fresh set and the old set according to a boundary *t*_*b*_ ∈ (*t*_*a*_, *t*_*c*_). If an edge was constructed in (*t*_*a*_, *t*_*b*_), it belongs to the old set otherwise the fresh set. The fraction of old edges and fresh edges are denoted as *p*_*older*_ and *p*_*fresher*_. The *p*_*fresher*_ can be comprehended as the observation length of historical information. Then, the popularity of node *i* is

2 $${s}_{i}=\frac{{\rm{\Delta }}{k}_{i}({t}_{b},{t}_{c}-{t}_{b})}{{\rm{\Delta }}{k}_{i}({t}_{a},{t}_{c}-{t}_{a})}=\frac{{k}_{i,fresher}}{{k}_{i,all}},$$

where *k*_*i,all*_ and *k*_*i,fresher*_ indicate the whole degree and fresher degree of node *i*. Equation (2) improves the drawbacks of simply counting the new edges and quantifies the popularity in the normalized range. Clearly, if all links of node *i* locate in the fresh set, *s*_*i*_ = 1. For another case that all links of node *i* locate in the old set, node *i* becomes dormant, *s*_*i*_ = 0. Therefore *s*_*i*_ ∈ [0, 1] and a higher *s*_*i*_ means a higher popularity.

### Popularity based structural perturbation method

In this section, we propose a hypothesis that the observed network is determined by some latent attractors (e.g. similar hobbies, ages, gender, location) that independently influence the structural properties. For an attractor ${x}_{k}={[{x}_{k,1},{x}_{k,2},\ldots ,{x}_{k,n}]}^{T}$, *﻿﻿x*_k,i﻿_﻿ represents the attractiveness of node *i* for the latent attractor *x*_*k*_. Inspired by configuration model, the probability *p*_*ij*_ that an edge exists between two node *i* and *j* is proportional to *x*_*k*,*i*_ *x*_*k*,*j*_. Supposing that there are *m* kinds of attractors, probability *p*_*ij*_ is defined as the weighted influence of each attractor,

3 $${p}_{ij}=\sum _{k=1}^{m}{w}_{k}{x}_{k,i}{x}_{k,j},$$

where *w*_*k*_ is a tunable parameter to balance the relative influence of each attractor *x*_*k*_. The problem is how to seek the optimal *w*_*k*_ and *x*_*k*,*i*_ that make *p*_*ij*_ approximate *a*_*ij*_ at most. Considering a network *G* with adjacent matrix *A* = (*a*_*ij*_)_*n* × *n*_, a special case is that *p*_*ij*_ = 1 if *a*_*ij*_ = 1, otherwise *p*_*ij*_ = 0. For optimal *w*_*k*_ and *x*_*k*_,

4 $${A}_{p}={({p}_{ij})}_{n\times n}=\sum _{k=1}^{m}{w}_{k}{x}_{k}{x}_{k}^{T}.$$

If *m* = *n* in Eq. (4), where *n* is the size of the network, then Eq. (4) could be comprehended as the matrix decomposition, with *w*_*k*_ and *x*_*k*_ representing eigenvalues and eigenvectors respectively. In practice, many random connections exist in networks, L*ü et al*. proposed the structural perturbation method (SPM) that can reduce the influence of randomness^28^. In SPM, a small fraction *p* ^*H*^ of edges Δ*A* is removed from the network, adjacent matrix *A* ^*R*^ of the remaining network is decomposed into

5 $${A}^{R}=\sum _{k=1}^{n}{\lambda }_{k}{x}_{k}{x}_{k}^{T},$$

where *λ*_*k*_ and *x*_*k*_ are the eigenvalues and eigenvectors of *A* ^*R*^, $|{x}_{k}|=1$. We could use *A* ^*R*^ to evaluate *A* with

6 $$\tilde{A}=\sum _{k=1}^{n}({\lambda }_{k}+{\rm{\Delta }}{\lambda }_{k})\,{x}_{k}{x}_{k}^{T},$$

where ${\rm{\Delta }}{\lambda }_{k}\approx \frac{{x}_{k}^{T}{\rm{\Delta }}A{x}_{k}}{{x}_{k}^{T}{x}_{k}}$ is the coupling influence of *x*_*k*_ on *λ*_*k*_. *Ã* actually is a special case of *A*_*p*_, (*λ*_k_ + Δ*λ*_*k*_) and elements of eigenvector *x*_*k*_ represent weight difference and the attractiveness for attractor *x*_*k*_ separately.

As we have argued, the ability for node *i* to attract new edges is determined by both latent attractors and its current popularity. To better meet practice, an advanced attractiveness ${x}_{k,i}^{^{\prime} }$ is proposed as

7 $${x}_{k,i}^{^{\prime} }={x}_{k,i}(1+\alpha {s}_{i}),$$

where *α* indicates the degree of temporal popularity. Equation (7), a combination of the static attractiveness and popularity, tightly captures both the static features and the temporal information of the evolving pattern. Later in Eq. (6), substituting *x*_*k*_ with *x′*_k_ to predict future links,

8 $$\mathop{{A}^{^{\prime} }}\limits^{ \sim }=\sum _{k=1}^{n}({\lambda }_{k}+{\rm{\Delta }}{\lambda }_{k}){x}_{k}^{^{\prime} }{x}_{k}^{^{\prime} T}.$$

Since Eq. (5) degenerates into Eq. (4) if the size *m* of attractors is less than *n*. Supposing that $|{\lambda }_{1}| > |{\lambda }_{2}| > \ldots  > |{\lambda }_{n}|$, we substitute *w*_*k*_ and *x*_*k*_ in Eq. (4) with *λ*_*k*_ and *x*_*k*_ in Eq. (5). Similar to the same transition from Eq. (5) to Eq. (8), we obtain

9 $$A^{\prime} ={({p}_{ij})}_{n\times n}=\sum _{k=1}^{m}({\lambda }_{k}+{\rm{\Delta }}{\lambda }_{k}){x}_{k}^{^{\prime} }{x}_{k}^{^{\prime} {{\rm T}}},$$

which reduces into Eq. (8) if *m* = *n*. In the following experiments, we firstly measure the performance of Eq. (8), then show that we could reduce the calculation complexity by using only a few eigenvalues and eigenvectors, that is $m\ll n$ in Eq. (9).

### Experiments on real networks

**Figure 2:**
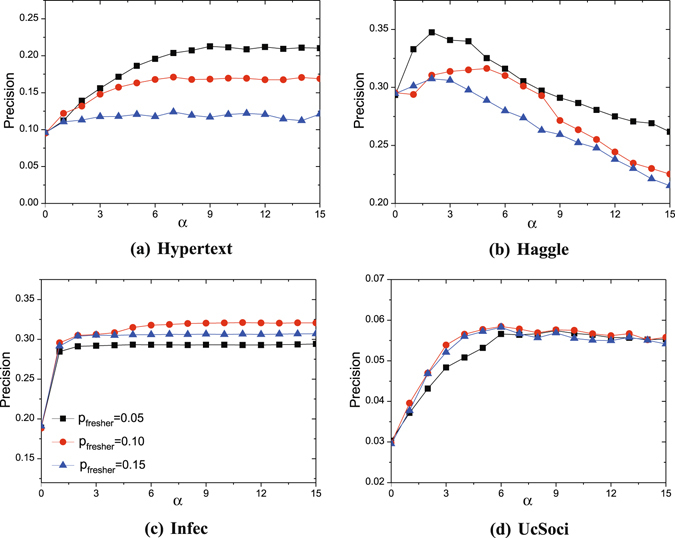
Precision versus *α* obtained by PBSPM. The experiments are performed on 90% training set and 10% probe set. Each data point is averaged over 10 independent realizations. The values of *p*_*fresher*_ and *α* corresponding to the optimal precision reported in Table 1 vary for different networks: 0.05 and 9 for Hypertext, 0.05 and 2 for Haggle, 0.10 and 11 for Infec, 0.10 and 7 for UcSoci.

The proposed method PBSPM, integrating the attractiveness *x*_*k*,*i*_ and popularity *s*_*i*_, reduces into the original SPM when *α* = 0. With the increase of *α*, PBSPM prefers to predict links between popular nodes. Figure 2 gives the performance of PBSPM in contrast to SPM (*α* = 0) under different *p*_*fresher*_. The precision values tend to be stable or achieve the best when *α* brings the static attractiveness and popularity into balance. Clearly, the optimal value of *α* varies for different networks. For Hypertext, Infec and UcScoci, future links have high likelihood to exist between the active nodes. However, for the Haggle dataset, the temporal trend of nodes are less obvious. Hence, the precision curve is optimized at *α* = 2, contrast to the other three networks of which the curves finally stabilize when *α* increases. Overall, when *α* ∈ [3, 5], PBSPM achieves improved performance compared with SPM in the four networks. Moreover, given the different length of historical information *p*_*fresher*_, all the curves present different levels of superiority in precision, suggesting a general and robust range of *p*_*fresher*_. Actually, it is difficult to choose the optimal value, which should follow the principle of keeping the balance between the length of historical information and future information (probe set). With regard to 10% probe set in this experiment, *P*_*fresher*_ = 0.1 is the balanced option because the corresponding curves all show the great improvements.

**Figure 3:**
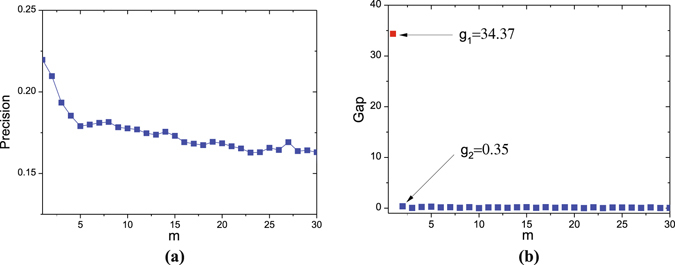
Precision versus *m* and gap ${g}_{m}=|{\lambda }_{m}|-|{\lambda }_{m+1}|$ for Hypertext. *λ*_*m*_ is the eigenvalue of adjacent matrix *A* ^*T*^. Panel (a) shows the performance of Eq. (9) on various *m* ∈ [1, 30] with fixed *p*_*fresher*_ = 0.05 and *α* = 9. Each data point is obtained over ten simulations. Panel (b) shows the difference *g*_*m*_ between $|{\lambda }_{m}|$ and $|{\lambda }_{m+1}|$. *g*_1_ = 34.37 is distinct and the others are all close to 0.

Reducing the number of eigenvectors could reduce the computation complexity. To address the high computation complexity, we propose the fast PBSPM that takes into account a few eigenvectors with only some large eigenvalues, which can well reflect the backbone structure of networks^33^. In practical networks, a huge gap exists in the eigenvalue space. Some eigenvectors with large eigenvalues play more important roles than those with small eigenvalues. Taking Hypertext as example, Fig. 3(a) plots the precision for various *m* in Eq. (9). Compared with SPM, the curve presents significant improvements and achieves the best at *m* = 1, meeting the effectiveness of Eq. (9). Figure 3(b) gives the differences between two adjacent eigenvalues ${g}_{m}=|{\lambda }_{m}|-|{\lambda }_{m+1}|$ $(|{\lambda }_{1}| > |{\lambda }_{2}| > \ldots  > |{\lambda }_{n}|).$ The distinct *g*_1_ indicates a huge gap between $|{\lambda }_{1}|$ and $|{\lambda }_{2}|$, while the other gaps (*m* ≥ 2) are all close to 0, suggesting that the huge gap *g*_1_ induces the decline of precision when *m* > 1. Then, we choose *m* = 1 as the optimal value for Hypertext, analogously, the values for Haggle, Infec and UcSoci are respectively determined as *m* = 2,19,2 after which the *g*_*m*_ approaches to 0 approximately. In consequence, it only requires *O*(*n* ^2^) time to calculate the top-*m* eigenvalues and corresponding eigenvectors, and the reconstruction of similarity matrix (Eq. 9) needs *O*(*m* × *n* ^2^) time. To reduce the randomness, the fast PBSPM repeats the random perturbation for ten times and obtains the averaged similarity matrix with *O*(10 × (*mn* ^2^ + *n* ^2^)) time. Hence, with $m\ll n$ and the increase in size *n*, the time complexity of fast PBSPM is *O*(*n* ^2^) in contrast with the time complexity *O*(*n* ^3^) of PBSPM and SPM, where the decomposition and reconstruction consume *O*(*n* ^3^) time. Besides, the time complexity is *O*(*n* ^2^) for local similarity based methods, such as CN, RA, AA, and *O*(*n* ^3^) for Katz and SRW.

Table 1 and Table 2 list the precision values and computation time of different link prediction algorithms. Obviously, the proposed methods achieve remarkable improvements, at most 84.84% for Hypertext, 28.42% for Haggle, 6.19% for Infec, 95.97% for UcSoci. In spite of this, PBSPM suffers from the huge computational cost that limits its extensive applications. Fast PBSPM, a well trade-off of computation complexity and accuracy, has the reasonable computational cost and the high accuracy. Due to the repeated steps in experimental procedures, the fast algorithm still consumes more time than some traditional predictors with the same time complexity. Additionally, the attractors ignored by the fast algorithm contain some secondary information that may either improve the accuracy as useful information or deteriorate the performance as network noise, hence, the precision slightly fluctuates around that of PBSPM. In general, the proposed methods show the high robustness because of the well performance for disparate networks, while other baselines give poor predictions for some networks. Apart from precision improvements, we also try to quantify the physical difference between the age of links selected by various methods, which can be comprehended as the average popularity of endpoints $\overline{s}=\sum \frac{{s}_{i}+{s}_{j}}{\mathrm{2\ast |}{E}^{P}|}$ if edge *e*_*ij*_ is selected by a certain predictor. According to Table 3, links selected by the proposed methods are much older than the others; that is, the potential links prefer to form between the active nodes in the earlier future.

**Table 1:** Precision of different methods for four networks. All the results are calculated under the optimal cases by adjusting parameters if any.

Precision	CN	AA	RA	Katz	SRW	SPM	PBSPM	Fast PBPSM
Hypertext	0.0959	0.1050	0.1005	0.0959	0.1187	0.0984	**0.2128**	**0.2194**
Haggle	0.1786	0.1888	0.1939	0.2041	0.2194	0.2928	**0.3475**	**0.3760**
Infec	0.0233	0.1163	0.1814	0.0233	0.3023	0.1949	**0.3210**	**0.3070**
UcSoci	0.0138	0.0153	0.0138	0.0138	0.0046	0.0298	**0.0584**	**0.0574**

The data in bold face are averaged over ten realizations with the same *p*_*fresher*_ and *α*.

**Table 2:** Computation time of different methods for four networks.

Time(*ms*)	CN	AA	RA	Katz	SRW	SPM	PBSPM	Fast PBPSM
Hypertext	1.02	1.12	1.08	1.51	1.95	20.3	**20.51**	**15.73**
Haggle	2.25	2.58	2.54	3.08	4.78	50.45	**51.62**	**28.86**
Infec	5.62	6.22	5.95	7.39	11.4	175.71	**179.15**	**92.8**
UcSoci	204.27	239.23	228.62	272.06	856.08	15200.76	**15902.53**	**1122.95**

All the results are averaged over ten runs on AMD R7 computer with MATLAB R2016b and 8GB RAM.

**Table 3:** Average age of links selected by predictors.

Precision	CN	AA	RA	Katz	SRW	SPM	PBSPM	Fast PBPSM
Hypertext	0.0411	0.0420	0.0445	0.0407	0.0509	0.0420	**0.2115**	**0.2243**
Haggle	0.0508	0.0513	0.0518	0.0488	0.0489	0.0609	**0.1313**	**0.1265**
Infec	0.0275	0.1228	0.2180	0.0275	0.4511	0.2148	**0.7901**	**0.8145**
UcSoci	0.0611	0.0666	0.0861	0.0617	0.1498	0.0672	**0.4407**	**0.4051**

The bold data are averaged over ten runs and obtained under the optimal parameters.

**Figure 4:**
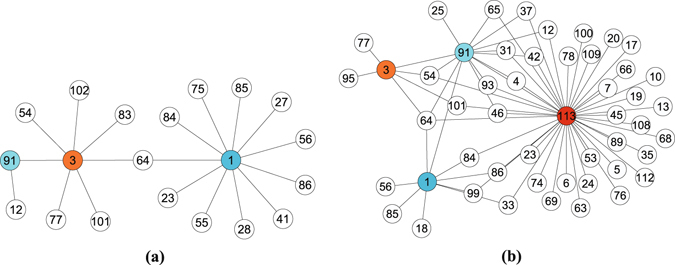
Predicted connections of large-degree node 1, 3 and active node 91, 113 in Hypertext. Only the selected nodes and their neighbors are plotted, and the connections are the subset of the top-$|{E}^{P}|$ predicted links. Panel (a) shows the connections predicted by SPM. Node 1 and 3 are much more attractive than node 91, and node 113 is not presented because of no connections. Panel (b) shows the connections predicted by PBSPM. The active node 91 and 113 attract numerous nodes, which gives rise to the explosive growth of edges.

In the following, we mainly focus on the performance of SPM and PBSPM to explore underlying reasons of the improvements. To figure out the effect of popularity, four typical nodes from the training set of Hypertext, the large-degree node 1 and 3 (*k*_1,*training*_ = 78,*s*_1_ = 0.051;*k*_3,*training*_ = 93,*s*_3_ = 0.032), and the active node 91 and 113 (*k*_91,*training*_ = 29,*s*_91_ = 0.289;*k*_*11*3,*training*_ = 14,*s*_113_ = 1) are chosen to analyse their predicted connections and corresponding variation of attractiveness. Figure 4 plots the predicted future links attached to selected nodes by SPM and PBSPM when *p*_*fresher*_ = 0.05 and *α* = 9. After that, the principal eigenvector *x*_1_ of *A* ^*R*^ and the advanced ${x}_{1}^{^{\prime} }$ under the optimal case are calculated to quantify the attractiveness for the most weighted attractor. In addition, the principal eigenvector also characterizes the ranking of nodes, i.e. the importance^34, 35^. In Fig. 4(a), node 1 and 3 (*x*_1,1_ = 0.1715,*x*_1,3_ = 0.1899) with the high importance are much more attractive than node 91 and 113 (*x*_1,91_ = 0.0648,*x*_1,113_ = 0.0329), especially, node 113 with the lowest importance has no connections at all. Contrastingly, the high popularity enhances the active nodes (${x}_{1,91}^{^{\prime} }=\mathrm{0.1158,}{x}_{1,113}^{^{\prime} }=0.1923$) and results in the burst of links connecting to the them in Fig. 4(b), notably the most active node 113. In summary, nodes with the higher popularity are emphasized by PBSPM to attract much more links, whereas the inactive despite their importance are weakened to reduce connections.

The above figures conduce to the understanding of how popularity imposes effects on several typical nodes, but note that, it is a rational speculation that the improvements must result from the advanced attractiveness of all nodes. As above argued, principal eigenvector denotes the attractiveness for the most weighted attractor. Because $({\lambda }_{1}+{\rm{\Delta }}{\lambda }_{1})({x}_{1}{{x}_{1}}^{T})$ occupies the main body of *Ã*, neglecting constant term Δ*λ*_1_ + Δ*λ*_1_, similarity *ã*_*i*,*j*_ is mainly determined by eigenvector *x*_1_. The Pearson correlation coefficient (*CC*) between principal eigenvector and degree in the probe set, holistically reflecting the extent to which the attractiveness *x*_1,*i*_ coincides with real degree increment *k*_*i*,*probe*_, is computed as follows,

10 $$cc=\frac{1}{n}\sum _{i=1}^{n}(\frac{{x}_{\mathrm{1,}i}-{\overline{x}}_{\mathrm{1,}i}}{{\delta }_{{x}_{\mathrm{1,}i}}})(\frac{{k}_{i,probe}-{\overline{k}}_{i,probe}}{{\delta }_{{k}_{i,probe}}}),$$

where ${\overline{x}}_{\mathrm{1,}i}$ and ${\overline{k}}_{i,probe}$ are the means of *x*_1,i_ and *k*_*i*,*probe*_. The *CC* between advanced ${x}_{1}^{^{\prime} }$ and degree in the probe set is obtained similarly. Table 4 lists the variation of *CC* after the addition of popularity and the coupling influence Δ*λ*_1_ averaged over ten independent perturbations. The positive Δ*CC* of four networks suggest attractiveness of some nodes are corrected to meet the degree increment in the future. Furthermore the positive Δ*λ*_1_ also strengthens the improvements of correlations. As a result, the popular nodes are assigned more connecting opportunities to promote the precision.

**Table 4:** Variation of correlation coefficient Δ*CC* and coupling influence Δ*λ*_1_.

Networks	Hypertext	Haggle	Infec	UcSoci
Δ*CC*	0.28	0.0582	0.3092	0.1155
Δ*λ*_1_	4.1822	4.79	1.86	4.2183

Each data is averaged over ten perturbations.

Eventually, to demonstrate the feasibility of the proposed methods in practical applications, we compare the fast PBSPM with time series (TS) based methods on continuous temporal networks, which have been effectively applied to the temporal link prediction^36–38^. For each network, the dataset is divided into *T*_*N*_ snapshots $({G}_{1},{G}_{2},\mathrm{...},{G}_{{T}_{N}})$ with the length of time period *P*_*length*_ = 7 days. Setting a specified time window *T* = 5, we use the graph series (*G*_*t*_, *G*_*t* + 1_, …, *G*_*t* + *T* − 1_) and its reduced static graph *G*_*t~t* + *T* − 1_ to predict the links that will occur in *G*_*t* + *T*_ (*t* = 1, 2, …, *T*_*N*_ − *T*). Then the popularity of each node is calculated as:

11 $${s}_{i}=\frac{{k}_{i,{G}_{t+T-1}}}{{k}_{i,{G}_{t \sim t+T-1}}}=\frac{{k}_{i,fresher}}{{k}_{i,all}}.$$

**Figure 5:**
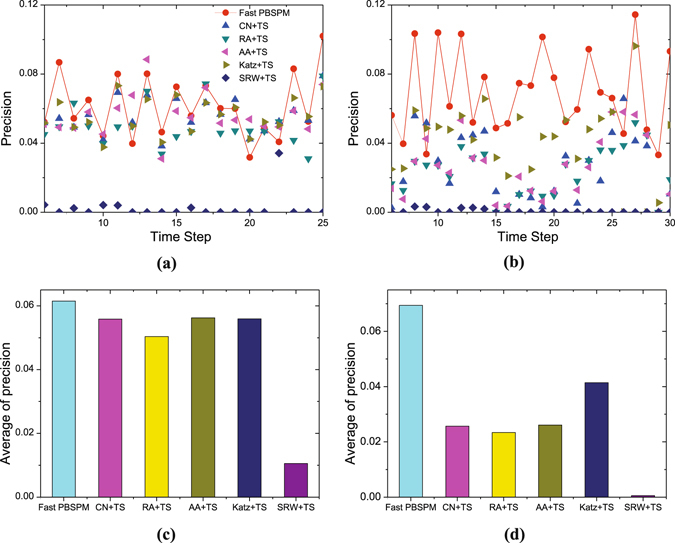
Precision at different time steps and their average values. *G*_*t~t* + *T* − 1_(*G*_*t*_, *G*_*t* + 1_, …, *G*_*t* + *T* − 1_) and *G*_*t* + *T*_ play the role of the training set *E* ^*T*^ and probe set *E* ^*P*^. Panel (a) and panel (b) show the precision values at different time steps for LKMLR and Wiki. The red curves are respectively obtained by the fast PBSPM with *α* = 2 and 5, *m* = 2 and 2. The other results are obtained under optimal cases by different forecasting models. Panel (c) and panel (d) give the average precision values of different methods for the two networks.

During the evolution, certain mechanisms drive the network organization regularly and the structural features keep relatively stable. Hence, we obtain the optimal *α* and *m* by the known networks observed between the time period 1 ≤ *t* ≤ 6 (*G*_1~5_ as the training set, *G*_*6*_ as the probe set) and apply them to the subsequent predictions. Figure 5 shows the precision at continuous time steps and the average accuracy of different methods. For LKMLR, though the fast PBSPM falls behind sometimes, its average value shows a slight advantage in precision (Fig. 5(a) (c)). For Wiki, not only does the fast PBSPM gain the upper hand at any time, but it achieves much higher average accuracy compared with TS based methods (Fig. 5(b) (d)). These experimental results demonstrate that the fast PBSPM has prospective applications in evolving networks.

## Discussion

In this paper, we propose the PBSPM and its fast algorithm to predict future links. The main contribution is to investigate the popularity (activeness) of nodes in real-world evolving networks and apply it to link prediction. Unlike previous works that calculate temporal effects with complex theories, we infer the popularity of each node by its recently active edges. Then we propose a hypothesis that the future network is influenced by both existing structure and popularity of nodes. By introducing popularity into perturbation method, PBSPM could distinguish active and inactive historical important nodes, and prefer to predict new edges attached to active nodes. Subsequently, the fast method is proposed to get rid of the high computation complexity. Experimental results on real-world evolving networks reveal that compared with traditional methods, the proposed methods achieve better performance in precision and robustness. Besides, further experiments are conducted to uncover the underlying reasons of the improvements.

Definitely, the performance of proposed methods largely depend on the popularity of each node. In other words, the popularity based methods are more applicable for the networks with obvious temporal effects, where the popularity metric can effectively quantify the popularity of each node. Hence, another important issue is that improving popularity performance would enhance the precision of link prediction, which is the future work. Since our work mainly explores prediction in evolving networks, it has possible applications in traffic prediction, airline control, recommendation of social network, and so on.

## Methods

### Experimental procedures

To predict the future links of evolving networks with PBSPM, there are five detailed steps to follow:

Step 1: We firstly divide the network into the training set *E* ^*T*^ and the probe set *E* ^*P*^ based on the birth time of each edge, the corresponding adjacent matrix are denoted by *A* ^*T*^ and *A* ^*P*^.

Step 2: The training set is further divided into the old set and the fresh set to calculate the popularity via Eq. (2) or Eq. (11).

Step 3: We perturb the training set by randomly removing a small fraction *p* ^*H*^ = 0.1 of edges Δ*A*, obviously, *A* ^*T*^ = *A* ^*R*^ + Δ*A*.

Step 4: We decompose the matrix *A* ^*R*^ and obtain the $\tilde{A^{\prime} }$ via Eq. (7) and Eq. (8).

Step 5: Repeat step 3 and step 4 for ten times. In other words, we implement the perturbations for ten times to obtain the averaged $\langle \tilde{A^{\prime} }\rangle$ where the score $\langle {\tilde{a^{\prime} }}_{ij}\rangle$ represents the existent likelihood of the link between node *i* and *j*. Finally, non-observed edges with the top-$|{E}^{P}|$ scores are chosen as potential future edges.

### Data description

In this work, six datasets are considered to evaluate the performance of algorithms. (1) Hypertext 2009 (Hypertext): a network of face-to-face contacts of the attendees of the ACM Hypertext 2009 conference from June 30 to July 1, 2009, including 113 nodes and 2196 unique links^39^. (2) Haggle: an undirected network representing contacts between people measured by carried wireless devices^40^, including 188 nodes and 1947 unique links. The time span is 4 days. (3) Infectious (Infec): a network describing the face-to-face behavior of people during the exhibition INFECTIOUS: STAY AWAY in 2009^39^, including 301 nodes and 2145 unique links. The time span is 8 hours. (4) UC Irvine messages (UcSoci): a directed network of messages between the users of an online community of students from the University of California, Irvine^41^, including 1692 nodes and 13037 unique links. The dataset spans from April 15 to October 25, 2004. (5) Linux kernel mailing list replies (LKMLR): a communication network of the Linux kernel mailing list. The data considered in experiments is from January to June, 2013, including 2907 nodes and 78955 links. (6) Wikipedia elections (Wiki): a network of users from the English Wikipedia that voted for and against each other in admin elections. The data considered in experiments spans from October, 2005 to April, 2006, including 2309 nodes and 23707 links^42^.

To simplified the problem, we ignore the direction and weighted of links, and remove the isolated nodes. What is more, the networks are divided into historical training set and future probe set only according to the timestamps that attach to edges.

### Evaluation metric

AUC (Area Under the receiver operating characteristic Curve) and Precision are two standard metrics used to measure the link prediction algorithm^43, 44^. The former randomly compares the score of a missing link with a non-existent link to evaluate the performance. The latter focuses on the links with top-*L* scores. When dealing with highly skewed datasets, the precision always gives a more informative picture of algorithms’ performance^45^. Hence, We choose Precision index as the metric to evaluate the accuracy of the proposed method and other baselines. Precision is defined as the ratio of links predicted accurately to all links selected. Namely if we select top-*L* links in the all ranked non-observed links and only *L*_*r*_ links are predicted correctly in the probe set *E* ^*P*^, then the accuracy of predictor follows

12 $${Precision}=\frac{{{L}}_{{r}}}{{L}}.$$

In our experiments, we select ${L}=|{{E}}^{{P}}|$ and count how many of top-$|{{E}}^{{P}}|$ links really exist in the probe set.

### Baselines

For comparison, we briefly introduce five traditional algorithms based on all three kinds of structural similarity.

1. Common Neighbors (CN), related to the concepts of the triadic closure, is the most well-known method with an assumption that two target points tend to connect with each other if the new connection may produce much more triangles in the graph.
  13 $${s}_{xy}^{CN}=|{\rm{\Gamma }}(x)\cap {\rm{\Gamma }}(y)|,$$
  where Γ(*x*) is the set of neighbors of node *x* and $|{\rm{\Gamma }}(x)\cap {\rm{\Gamma }}(y)|$ represents the set of common neighbors of *x* and *y*.
2. Adamin-Adar (AA), advanced from CN, restricts the contributions of common neighbors by introducing a penalty factor, i.e., the logarithm of reciprocal of their degree.
  14 $${s}_{xy}^{AA}=\sum _{z\in {\rm{\Gamma }}(x)\cap {\rm{\Gamma }}(y)}\frac{1}{\mathrm{log}\,{k}_{z}},$$
  where *k*_*z*_ denotes the degree of common neighbor *z*.
3. Resource Allocation (RA), motivated by transferring resource between two unconnected nodes, views the common neighbor as the intermediary of which the transfer capability equals to the reciprocal of degree of common neighbors.
  15 $${s}_{xy}^{RA}=\sum _{z\in {\rm{\Gamma }}(x)\cap {\rm{\Gamma }}(y)}\frac{1}{{k}_{z}}.$$
4. Katz index, based on global information of network, counts all the paths connecting two endpoints with weakening the contributions of longer paths exponentially:
  16 $${s}_{xy}^{Katz}=\sum _{l=1}^{\infty }{\alpha }^{l}\cdot |path{s}_{x,y}^{\langle l\rangle }|.$$
  When $|\alpha | < 1/{\lambda }_{{\rm{\max }}}$, it can be rewritten as:
  17 $$S={(I-\alpha \cdot A)}^{-1}-I,$$
  where *I* is the identity matrix, *α* > 0 is the tunable parameter, *λ*_max_ is the largest eigenvalue of the adjacent matrix *A*.
5. Superposed Random Walk (SRW) considers the summation of local random walks within *t* steps and degree of two endpoints to emphasize the local properties in real networks^26^.
  18 $${s}_{xy}^{SRW}(t)=\sum _{\tau =1}^{t}[{q}_{x}{\pi }_{xy}(\tau )+{q}_{y}{\pi }_{xy}(\tau )],$$
  where ${q}_{x}=\frac{{k}_{x}}{2|E|}$ denotes the initial distribution of resources and *π*_*xy*_(*τ*) represents the transfer probability from *x* to *y*.
6. Time series based methods explore the evolution of topological metrics to predict the future links^37^. It follows the steps below:

Step1: Choose a static-structure method (e.g. CN, RA, Katz, etc);

Step2: Establish the time series by calculating the similarity between unconnected nodes in each time period;

Step3: Compute the final score of unconnected nodes with a forecasting model (e.g. Moving Average, Liner Regression, Simple Exponential Smoothing, etc);

Step4: Measure the algorithms with future links in the next time period.
